# Special Focus Issue on Emerging Scientists and Science Communication

**DOI:** 10.1089/dna.2021.29019.gbp

**Published:** 2022-01-12

**Authors:** Giorgia Brambilla Pisoni, Julia Noack, Silvia Preite

**Affiliations:** ^1^ Department of Global Health and Development, London School of Hygiene and Tropical Medicine, London, United Kingdom.; ^2^ Department of Microbiology and Immunology, University of California, San Francisco, California, USA.; ^3^ Gothenburg, Sweden.

Dear Readers,

We are three colleagues and friends who received the honor of curating the author's panel for this *DNA and Cell Biology* Special Focus Issue on Emerging Scientists and Science communication.

The current COVID-19 pandemic triggered our motivation to collaborate with *DNA and Cell Biology* to arrange this issue. We all felt the urgency to improve how the scientific world shares research findings with the public.

The issue results from a cross-disciplinary effort of young professionals who wish to engage us through short opinion articles disclosing their vision and informed scientific interests. In this study, we showcase the many flavors of science through a representative author panel that aims to disseminate knowledge from different research areas and scientific professions.

This collection contains 14 essays divided into four thematic sections:

1. Science policy, communication, and the relationship between art and science
2. Infectious diseases, including SARS-CoV2: pathogenesis, immune responses, vaccines, diagnostics, and broad impact on society
3. Scientific technologies to ease and advance basic and translational research
4. Environmental health: the relationship between people and the environment.

For science to be responsible and effective, the process of science communication must occur not only within scientists but also between scientists and the lay audiences. We are all testimonies of how science is becoming more and more central to society. Hence, better communication is necessary to build trust and lead to conscious actions, as individual decisions ultimately influence society.

We sincerely hope you will enjoy the following joint discussion about the current transformative era we are living in, in terms of scientific technology, ethics, interdisciplinarity, and inclusiveness.

With that, we wish not to delay your leafing through any further, and we leave you with a concise authors' description before reading their works.

Enjoy!

